# A multiplexed marker-based algorithm for diagnosis of carcinoma of unknown primary using circulating tumor cells

**DOI:** 10.18632/oncotarget.6657

**Published:** 2015-12-18

**Authors:** Elizabeth M. Matthew, Lanlan Zhou, Zhaohai Yang, David T. Dicker, Sheldon L. Holder, Bora Lim, Ramdane Harouaka, Si-Yang Zheng, Joseph J. Drabick, Nicholas E. Lamparella, Cristina I. Truica, Wafik S. El-Deiry

**Affiliations:** ^1^ Laboratory of Translational Oncology and Experimental Cancer Therapeutics, Division of Hematology-Oncology, Penn State Hershey Cancer Institute, Hershey, PA, USA; ^2^ Laboratory of Translational Oncology and Experimental Cancer Therapeutics, Department of Medical Oncology and Molecular Therapeutics Program, Fox Chase Cancer Center, Philadelphia, PA, USA; ^3^ Department of Pathology, Penn State Milton S. Hershey Medical Center, Hershey, PA, USA; ^4^ Division of Hematology-Oncology, Penn State Milton S. Hershey Medical Center and Penn State Hershey Cancer Institute, Hershey, PA, USA; ^5^ Department of Biomedical Engineering, Penn State University, University Park, PA, USA; ^6^ The University of Texas MD Anderson Cancer Center, Houston, TX, USA

**Keywords:** circulating tumor cells (CTCs), carcinoma of unknown primary (CUP), immunofluorescence, diagnosis

## Abstract

Real-time, single-cell multiplex immunophenotyping of circulating tumor cells (CTCs) is hypothesized to inform diagnosis of tissue of origin in patients with carcinoma of unknown primary (CUP). In 20 to 50% of CUP patients, the primary site remains unidentified, presenting a challenge for clinicians in diagnosis and treatment. We developed a post-CellSearch CTC assay using multiplexed Q-dot or DyLight conjugated antibodies with the goal of detecting multiple markers in single cells within a CTC population. We adapted our approach to size-based CTC enrichment protocols for capturing CTCs and subsequent immunofluorescence (IF) using a minimal set of markers to predict the primary sites for common metastatic tumors. The carcinomas are characterized with cytokeratin 7 (CK7), cytokeratin 20 (CK20), thyroid transcription factor 1 (TTF-1), estrogen receptor (ER) or prostate-specific antigen (PSA. IF has been optimized in cultured tumor cells with individual antibodies, then with conjugated antibodies to form a multiplex antibody set. With IF, we evaluated antibodies specific to these 5 markers in lung, breast, colorectal, and prostate cancer cell lines and blood from metastatic prostate and breast cancer patients. This advanced technology provides a noninvasive, diagnostic blood test as an adjunct to routine tissue biopsy. Its further implementation requires prospective clinical testing.

## INTRODUCTION

Metastasis is responsible for the majority of cancer-related deaths [1]. A limiting step in metastasis is access to circulation, thus theoretically circulating tumor cells (CTCs) should be present in all patients with metastatic tumors, which may have both diagnostic and prognostic values. Enumerated by the Food and Drug Administration-cleared Janssen (Veridex) CellSearch® system, circulating tumor cells are an independent prognostic factor of progression-free survival (PFS) and overall survival in metastatic breast [2-4], colon [5] and prostate cancers [6]. The technology is currently being used in the clinic to monitor disease progression as well as the response to cancer therapy. There is intense effort in the field to identify prognostic as well as predictive biomarkers in CTCs [7, 8].

Carcinoma of unknown primary (CUP) accounts for only 3-5% of metastatic tumors and presents a challenge for clinicians in diagnosis and therapy [9]. CUP patients can have early and unusually aggressive metastatic dissemination without a readily identifiable primary tumor site [9, 10]. In 20 to 50% of CUP patients, the primary site remains unidentified even after extensive diagnostic workup. Patients with CUP have a median survival of 8-11 months and a one-year survival of only 25% [9]. Tissue biopsy is the standard of care for diagnosis, but there is a need for improved and complementary methodology [9]. A non-invasive blood test that takes advantage of CTC analysis may be a valuable adjunct to routine biopsy.

Quantum dots (Q-dots) are nanocrystal fluorophores with long-term photostability and improved brightness, which allow simultaneous excitation of multiple fluorescence colors [11, 12]. DyLight fluorescent dyes are high-intensity, photostable fluorescent tags for labeling antibodies and other molecular probes. We have been developing a CTC analysis method using multiplexed conjugated antibodies with the goal of detecting multiple markers in each single cell within a CTC population to aid in the diagnosis of CUP and identification of its site of origin.

Isolation of circulating tumor cells involves both selection and enrichment. The cell surface marker most commonly utilized for isolation of CTCs is the epithelial cell adhesion marker (EpCAM). The Janssen (previously Veridex) CellSearch system with the EpCAM kit uses EpCAM-antibody bound ferrofluid to capture EpCAM positive cells with the use of a magnet. The cells are permeabilized and subjected to further positive selection by immunofluorescence with DAPI, antibody staining with Cytokeratins 8, 18 and 19 followed by negative selection with CD45 antibody staining [13-15].

The Janssen CellSearch is utilized in this study to diagnose carcinoma based on the presence of CTCs as described above and subsequent enumeration. CTCs are also enriched and captured based on their size utilizing either the flexible micro spring array (FMSA) device [16, 17] or the Creatv Microtech CellSieve device. The CTCs may be fixed and stained for multiple markers directly on either the FMSA device or the Creatv Microtech device.

Cytokeratin 7 (CK7), cytokeratin 20 (CK20), thyroid transcription factor 1 (TTF-1), estrogen receptor (ER) and prostate-specific antigen (PSA) are markers commonly used by pathologists in determining the site of origin from tissue biopsies [9, 18-24]. TTF-1 is a marker specific to the lung and thyroid [20], PSA is specific to prostate [22, 23] and ER to breast [9] and ovaries [21]. CK20 is particularly useful in diagnosing lower gastrointestinal carcinomas, whereas CK7 is expressed more commonly in upper gastrointestinal including pancreaticobiliary, respiratory and gynecological malignancies [9, 18, 21]. Our study utilizes these markers in CTCs to demonstrate the feasibility of differentiating breast and prostate cancer from other common metastatic tumors such as lung and colon cancers [25-27].

## RESULTS

### Marker-based algorithm for CTCs

**Figure 1:**
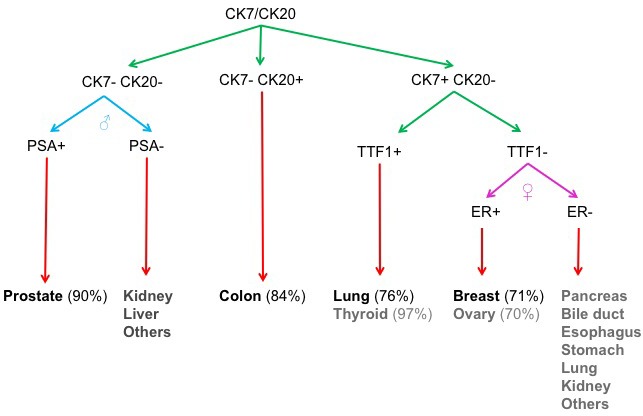
Minimal set of markers for carcinoma identification Four of the most common carcinomas (prostate, breast, colon and lung) are identified by immunofluorescence determination of the presence or absence of each of four out of five marker proteins. Cytokeratin 7 (CK7), cytokeratin 20 (CK20), thyroid transcription factor 1 (TTF-1), estrogen receptor (ER) in females or prostate-specific antigen (PSA) in males comprise the minimal set of markers for this algorithm. The percentages indicate the frequency of the tumor type showing positive staining for the markers.

**Figure 2:**
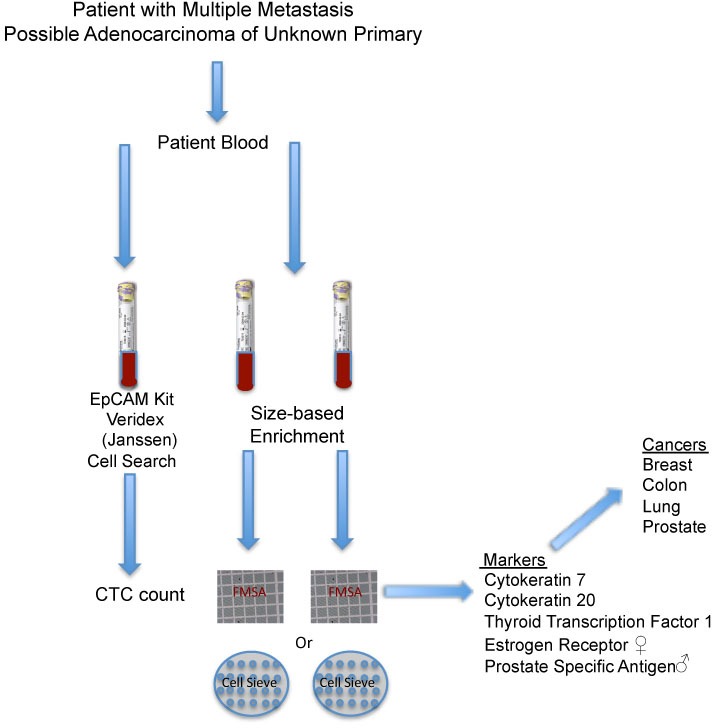
Clinical blood test for carcinoma of unknown primary A patient's blood is tested with the CellSearch (Veridex now Janssen) epithelial or carcinoma kit (using EpCAM), and the presence of CTCs, which are enumerated supports a diagnosis of carcinoma. Second and third tubes of blood are enriched for CTCs based on size using the FMSA device or CellSieve (Creatv Micro Tech) device followed by immunofluorescence (IF) analysis. IF evaluation of captured CTCs detects the presence of 4 of 5 marker proteins to distinguish between prostate, breast, colon and lung cancer.

We have developed an immunofluorescence (IF) protocol with a minimal set of markers to predict the primary sites for common metastatic tumors from lung, colon, breast or prostate cancer (Figure 1), and this algorithm may be expanded to include additional cancer types (Supplemental Figure S1). A patient's blood is tested using the Janssen (Veridex) CellSearch system and positive CTCs from the epithelial kit support a diagnosis of carcinoma. To identify the tissue origin of the carcinoma, we use cytokeratin 7 (CK7), cytokeratin 20 (CK20), thyroid transcription factor 1 (TTF-1), estrogen receptor (ER) in female patients or prostate-specific antigen (PSA) in male patients as the minimal set of markers (Figure 2).

### Marker verification process

**Figure 3:**
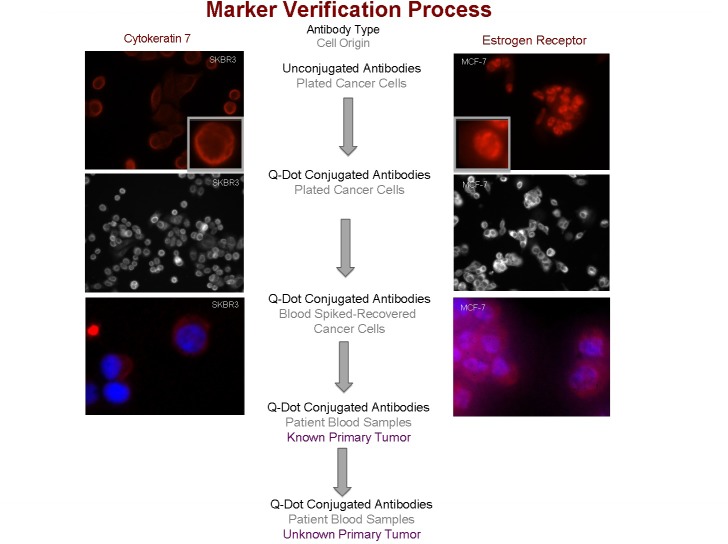
Marker validation process All five marker proteins (cytokeratin 7, cytokeratin 20, thyroid transcription factor, estrogen receptor and prostate-specific antigen) are validated using Step 1) unconjugated primary and fluorescent secondary antibodies in plated cells, Step 2) Q-dot conjugated primary antibodies in plated cells, Step 3) Q-dot conjugated primary antibodies in cells spiked into blood post-Veridex, Step 4) Q-dot conjugated antibodies in cells from cancer patient with known primary, Step 5) Q-dot conjugated antibodies in cells from cancer patients with unknown primary. The schematic demonstrates the first three steps in the process with DAPI nuclear stain included in the third panel.

The minimal set of markers we have chosen are commonly used by pathologists in identifying the primary site from tissue biopsies [9]. The antibodies used in paraffin-embedded or frozen tissue sections may or may not work in populations of individual cells evaluated by immunofluorescence. Therefore, each of the chosen primary antibodies was initially evaluated in cancer cell lines expected to be positive (Figure 3, top panels) or negative for the marker. When the chosen primary antibody coupled with a fluorescence-labeled secondary antibody was successful as defined by appropriate positive and negative staining in respective plated cancer cell lines, the primary antibody was subsequently conjugated to a Q-Dot or DyLight fluorophore. The conjugated primary antibody was re-evaluated in the respective cancer cell lines positive (Figure 3, middle panels) and negative for the target marker. The cell lines were subsequently spiked into human blood and processed through the Janssen (Veridex) Cell Search system. The cells recovered from blood are evaluated post-CellSearch with the Q-Dot conjugated antibody against that marker protein (Figure 3, bottom panels). Alternatively, Q-Dot and DyLight conjugated antibodies were used to stain the CTCs captured on FMSA or similar devices. Finally, CTCs from patients with known carcinomas were evaluated with Q-dot or DyLight conjugated antibodies after FMSA, CellSieve or similar enrichment or alternatively post-CellSearch. If the recovered circulating tumor cells show an immunophenotype consistent with the known primary, the future phase of the study is to prospectively evaluate patients with carcinomas of unknown primary. Figure 3 demonstrates the entire marker validation process with images of cell lines positive for either CK7 (left panels) or estrogen receptor (right panels).

### Marker verification of individual antibodies

**Figure 4:**
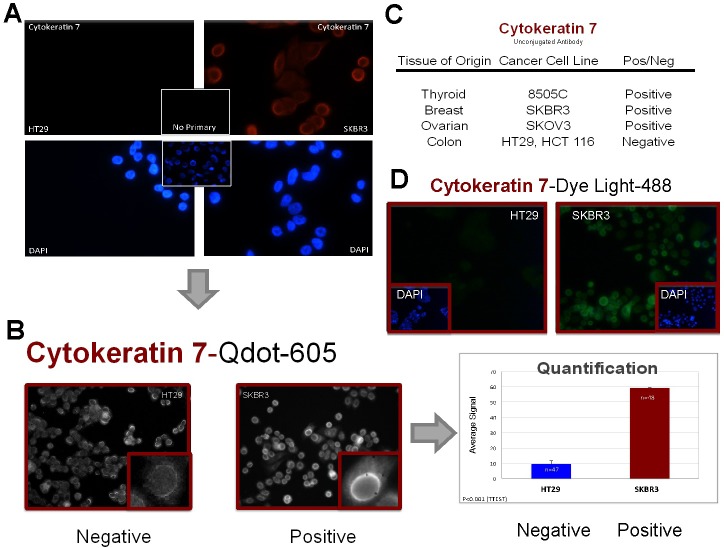
Cytokeratin 7 marker validation Cytokeratin 7 (CK7) demonstrates positive expression in SKBR3 cells but negative HT29 colon cancer cells. **A.** Unconjugated CK7 antibody. **B.** Q-Dot 605 conjugated CK7 antibody. Quantification: Regions of interest (47 to 48) around cells was utilized to calculate average signal intensity. **C.** CK7 expression in all the positive and negative cell lines evaluated with the unconjugated primary antibody. **D.** DyLight 488 conjugated to CK7 antibody after multiplexing with CK20-DyLight 594 (CK20 not shown).

**Figure 5:**
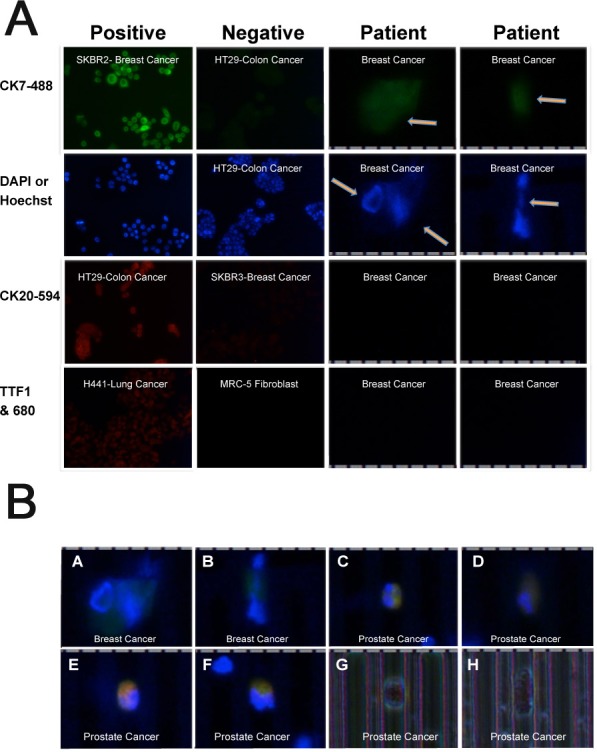
Metastatic breast and prostate cancer patient-derived CTCs Top Panel: Cytokeratin 7 (CK7), 4′,6-diamidino-2-phenylindole (DAPI) or Hoechst 33342, Cytokeratin 20 (CK20) and Thyroid transcription factor 1 (TTF1) expression in positive and negative control cancer cells (lower power) and breast cancer patient CTCs. CK7 is conjugated to DyLight 488; CK20 is conjugated to DyLight 594; TTF1 is used with Alexa Fluor 680 secondary antibody. Ruler with 10μm divisions below patient CTCs shown in region of 600x image. Experimental protocol shown in Supplementary Figure 9. Bottom Panel: **A.**-**B.** CK7 (green) and Hoechst (blue) positive cells from a metastatic breast cancer patient (Figure 5). **C.**-**F.** PSA (red), CK7 (green) and Hoechst (blue) merged image of cells from a metastatic prostate cancer patient. G) Phase image of cell in panel C. H) Phase image of cell in panel D. Ruler with 10μm divisions above images of patient CTCs. Images (600x): Zoom into region of image with CTC.

**Figure 6:**
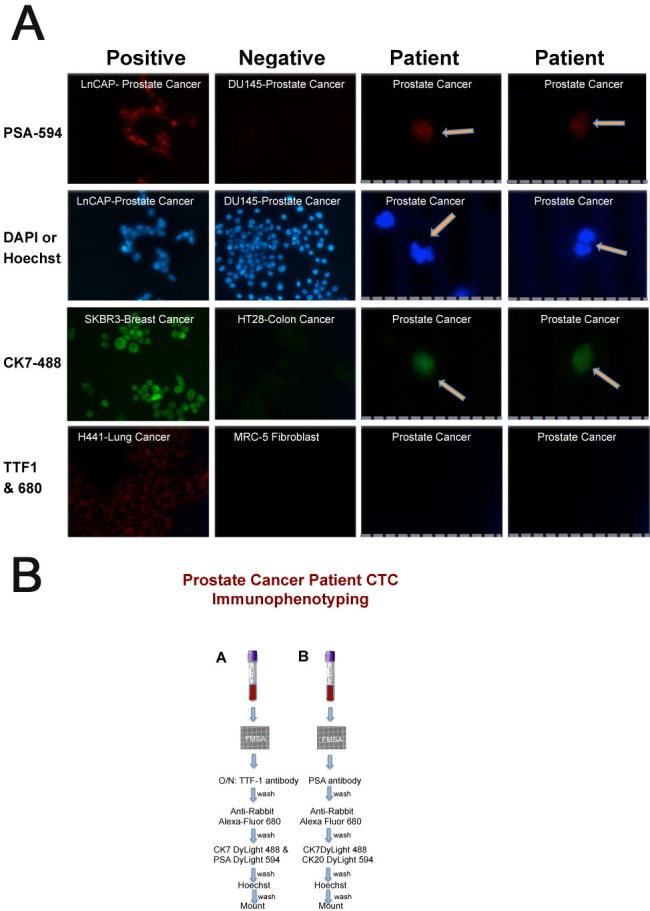
Metastatic prostate cancer patient-derived CTCs **A.** Prostate Specific Antigen (PSA), 4′,6-diamidino-2-phenylindole (DAPI) or Hoechst 33342, Cytokeratin 7 (CK7) and Thyroid transcription factor 1 (TTF1) expression in positive and negative control cancer cells and patient CTCs. PSA is conjugated to DyLight 594; CK7 is conjugated to DyLight 488; TTF1 is used with Alexa Fluor 680 secondary antibody. Ruler with 10μm divisions at the bottom of patient CTC images. **B.** Two tubes of blood were evaluated for IF after capture on the FMSA device as shown. The results from the first tube (tube A) are shown in panel A above and Figure 7C-7H.

Figure 4 demonstrates marker validation with a CK7 antibody that was subsequently conjugated to Q-Dot 605. As predicted by the algorithm with a minimal set of markers (Figure 1), SKBR3 breast cancer cells demonstrate positive staining of CK7 while HT29 colon cancer cells show no expression. The corresponding table summarizes the results from all the cells types tested. As expected, 8505C thyroid cancer cells and SKOV3 ovarian cancer cells demonstrate positive CK7 staining; another colon cancer cell line, HCT 116 was negative. Q-Dot conjugated antibodies are known to have some non-specific binding based on the presence of some weaker signal in the negative controls (Figure 4B, left panel). We were able to distinguish between positive (SKBR3) and negative (HT29) cell types both visually and through quantification of the average signal intensity. The CK7 signal in SKBR3 cells was approximately 6-fold stronger than that in HT29 cells. Using Nuance software, signal intensity in the area of interest was evaluated after subtracting the background and manually adjusting the threshold intensity. Although the majority of the areas of interest contained single cells, some had clumps of cells. Therefore, the n-value in the bar graph may be lower than the actual number of cells when clumps of cells were included in the area of interest. As an alternative to quantum dots, conjugating the CK7 antibody to DyLight 488 yielded minimal non-specific binding (Figure 4D) in a multiplexing experiment with CK20-DyLight 594. Supplemental Figure S2 demonstrates IF on cells captured on either the FMSA (CK7 conjugated to Q-Dot 605) or CellSieve devices after spiking SKBR3 cancer cells into normal donor blood. Antibodies against other markers in our minimal set of markers (Figure 1) such as CK20 (Supplemental Figure S3; Figure 5), ER (Supplemental Figure S4), PSA (Supplemental Figure S5; Figure 6) and TTF-1 (Supplemental Figure S6; Figures 5 & 6) that are suitable for cell IF have been identified. In addition, antibodies to other markers such as alpha-fetoprotein (AFP) for hepatocellular carcinoma (Supplemental Figure S7) and carcinoembryonic antigen (CEA) for colorectal cancer and some other epithelial cancers (Supplemental Figure S8) were identified for the purpose of characterizing a larger set of carcinomas.

### Optimizing CEA Q-Dot 655 in strongly and weakly positive cells

CEA is a secreted cell-surface glycoprotein, which is not included in our algorithm due to its poor tissue specificity. Using an unconjugated primary antibody and fluorescence-labeled secondary antibody, CEA expression was found to be positive in HT29 and weakly positive in HCT 116 colon cancer cells. Specifically, HCT 116 cells have lower levels of CEA and in some cells the expression levels were barely detectable. When the CEA antibody was conjugated to Q-Dot 655, these differences were more subtle, which is attributed to non-specific binding (Supplemental Figure 8). To increase the chance of detecting these differences, we optimized our Q-Dot IF protocol. Reducing the incubation time of the primary antibody and using different blocking reagents allowed differential detection of CEA expression between the strongly positive HT29 and weakly positive HCT 116 colon cancer cells (Supplemental Figure S9), which more closely matched the results obtained with unconjugated primary antibody and fluorescence-labeled secondary antibody (Supplemental Figure S8). However, application of this approach to other Q-Dot conjugated antibodies did not yield definitive results (data not shown) as seen with DyLight conjugated antibodies. In Figure 4D, the cells negative for CK7 (HT29) could be clearly distinguished from positive cells without quantification when CK7 antibody was conjugated to DyLight 488.

### Multi-marker algorithm-based analysis of patient CTCs

To further confirm this, we recruited a patient with ER-negative breast cancer based on prior pathology who had bone metastasis and positive CTCs (2 cells) by CellSearch analysis. Two additional tubes (∼7.5 mL each) of blood were enriched for CTCs with two FMSA devices. The results from one of the tubes showed at least 2 CK7-positive, Hoechst-positive, CK20-negative, TTF1-negative cells in the blood consistent with her breast cancer diagnosis (Figure 5 Top panel and Bottom Panel A-B; Supplemental Figure S10 tube A).

We also recruited a patient with castrate-resistant prostate cancer metastatic to both bone and meninges. Three weeks after demonstrating a serum PSA level of close to 1500 ng/mL, and one week after androgen biosynthesis inhibitor treatment, the patient had blood drawn for CTC enrichment and immunophenotyping. The first tube (∼7.5mL) of blood was evaluated with antibodies to TTF-1, CK7, PSA (and Hoechst) whereas the second tube was evaluated for PSA, CK7, CK20 (and Hoechst) as shown in Figure 6B. Results from the first tube of blood are shown side-by-side with cancer cells that are positive and negative for each of the markers, respectively (Figure 6A). Two CTCs that are TTF-1-negative, PSA-positive, CK7-positive with Hoechst-stained nuclei are shown in the last two columns. The CTC shown in the second column from the right is shown again in Figure 5F in a merged image of TTF-1 (negative), PSA and CK7. Although the majority of prostate cancer cells are CK7-negative, some do express CK7 (Figure 1) [28, 29]. The observed expression of PSA and absence of TTF-1 in Hoechst-stained nuclei are consistent with prostate cancer. Collectively these CTCs enriched from this patient's blood identify the patient's cancer type as prostate. Merged images of negative TTF-1, positive PSA and CK7 expression and Hoechst nuclear stain in three additional CTCs are shown in Figure 5 (Bottom Panel C, D & E). Phase microscopy of the cells in panel C and D are shown in panels G and H respectively with their oval shape on the FMSA device.

**Figure 7:**
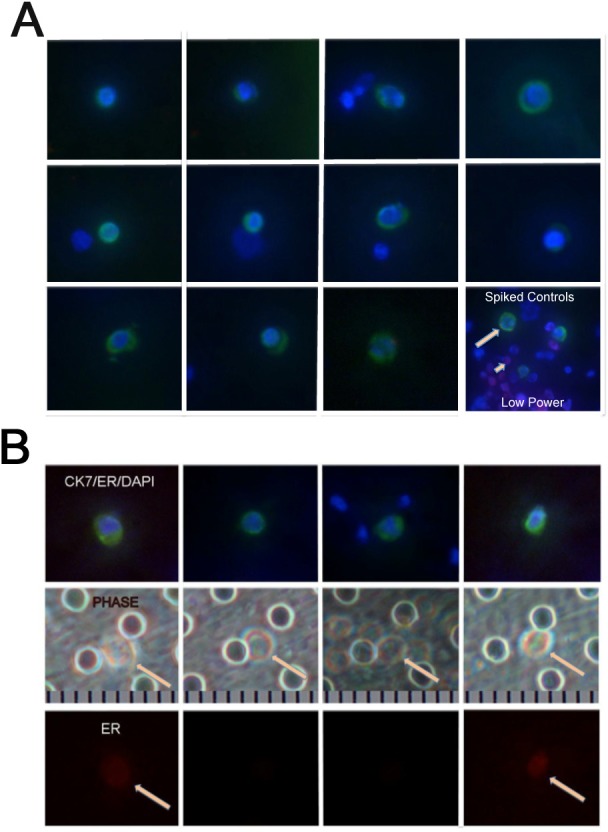
Metastatic breast cancer patient with elevated CTC count **A.** High power sampling of 11 CTCs captured from the blood of an Estrogen receptor (ER)/Progesterone-receptor positive metastatic breast cancer patient showing no ER expression. Merged images of Cytokeratin 7 (CK7)/Alexa Fluor 488 (green), ER/Cy3 (red, absent) and Hoechst 33342 nuclear stain (blue). Bottom right panel: positive control MCF7 cells (red, ER, short arrow) and SKBR3 cells (green, CK7, long arrow) with Hoechst 33342 nuclear stain (blue) after spiking into normal donor blood. **B.** High power images of CTCs captured during second blood draw. *Top Panels:* High power images of 4 different CTCs. Merged images of Cytokeratin 7/Alexa Fluor 488 (green), ER/Cy3 (red) and Hoechst 33342 nuclear stain (blue). *Middle Panels:* Phase microscopy of the same fields in panels directly above. Arrows point to CTCs. Ruler at the bottom: 10μm divisions. *Bottom panels:* ER expression alone of the same field as the panels directly above. Arrows point to ER-positive cells.

**Figure 8:**
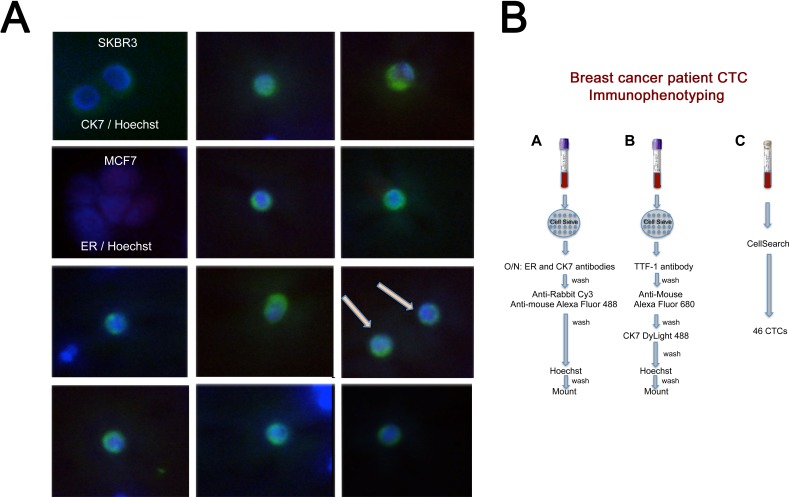
Metastatic breast cancer patient with elevated CTC count High power images of unique CTCs captured during second blood draw. Merged images of Cytokeratin 7 (CK7)/Alexa Fluor 488 (green), ER/Cy3 (red, absent) and Hoechst 33342 nuclear stain (blue). Far left column, top 2 panels: CK7 and ER positive control plated cells with Hoechst nuclear stain. **B.** Experimental protocol for results shown above. 167 CTCs by CellSearch during first blood draw (Figure 7) and 46 CTCs by CellSearch during second blood draw (Panel A above).

CTCs were captured from a second breast cancer patient with ER-positive, progesterone receptor (PR)-positive, human-epidermal growth factor receptor 2 (HER2)-negative breast cancer. This patient had bone metastasis and was treated with an aromatase (an enzyme in estrogen synthesis pathway)-inhibitor plus a mammalian target of rapamycin (mTOR) inhibitor. She had 38 CTCs by the clinical CellSearch test. She had blood drawn for this study 37 days later (referred to as the first IF/on-study blood draw) with 167 CTCs by CellSearch (Figure 7A) and subsequently another 26 days later (second IF/on-study blood draw) with 46 CTCs by the CellSearch method (Figure 7B and Figure 8). Two additional tubes of blood were collected each time on the first and second IF/on-study blood draws for enrichment with the CellSieve device and IF analysis (Figures 7 and 8) as shown in Figure 8B. Eleven of the first thirteen CK7-positive/Hoechst-positive cells seen in a high power sampling of the CTCs captured on the Cell Sieve device during the first IF blood draw are shown in Figure 7A as ER-negative. MCF7 (ER positive control) and SKBR3 (CK7 positive control) cells were spiked into normal donor blood and captured on the CellSieve device the same day as the patient sample (Figure 7A bottom right panel). Figure 8B shows the experimental schematic with results from tube A shown in Figures 7 and 8A. Automated low power images of the entire CellSieve device were captured at the Penn State Imaging Core. A manual count of the resulting composite image yielded 164 CK7/Hoechst-positive cells, which is notably similar to the 167 CTCs enumerated by CellSearch from a different tube of blood harvested at the same time. None of these cells had detectable ER expression. Since ER expression was seen in the spiked positive control MCF7 cells treated simultaneously with the patient sample, the absence of ER signal is highly unlikely to be an experimental error. To confirm and track ER loss, a second blood draw was performed. Although CellSearch identified 46 CTCs on the patient's second IF/on-study blood draw, by low power sampling (150x), we counted 69 CK7-positive/Hoechst-positive cells, 15 (21.7%) of which were ER-positive. The most recent bone marrow biopsy in 2012 revealed that approximately 5% of the metastatic cancer cells were ER positive, thus it is not surprising that an earlier sampling of circulating tumor cells yielded ER-negative CTCs. An additional 16 ER-positive/Hoechst-positive/CK7-negative cells were observed. It is possible these cells may have lost CK7 although this is infrequent. At high power (600x), we imaged at least 74 CK7-positive/Hoechst-positive cells with variable ER expression captured on this CellSieve device. Four of these cells are shown in Figure 7B with merged images of CK7/Hoechst/ER in the top panels, phase images of the same viable cells/field in the middle panels and ER expression alone of the same cells/field in the bottom panel. Another 11 CK7-positive/Hoechst-positive cells with variable ER expression are shown in Figure 8A with additional cells shown in Supplemental Figures S11 and S12. Cells captured on the CellSieve device from the second tube of blood (tube B; Figure 8B) showed no significant CK20 expression or definite TTF-1 expression. The absence of TTF-1 expression in the patient sample during the second blood draw could not be rigorously confirmed due to the absence of nuclear TTF-1 signal in H441 cells tested on the same day as the patient sample. All other positive and negative controls were appropriate. The large number of CK7/Hoechst-positive cells with or without ER expression on the first CellSieve device (from blood tube A) and presence of CK7/Hoechst-positive cells but no CK20 (and possibly no TTF-1) on the second CellSieve device (from blood tube B) of the patient after both on-study blood draws is consistent with the patient's known primary breast cancer.

## DISCUSSION

Our study established a new algorithm-based protocol, which enabled determination of the tissue of origin in patients with breast and prostate cancers, two of the most common metastatic cancers. Our proof-of-principle study showed that applying our algorithm in multiplex IF analysis of individual CTCs successfully identified the primary tumor in metastatic breast and prostate cancer patients. Future studies with more patients of known metastatic carcinomas (lung, colon, prostate, and breast) as well as CUP patients will pave the way for clinical application of this diagnostic blood test.

After evaluating different commercially available antibodies to CK7, CK20, TTF-1, ER and PSA, we have identified antibodies, which work well in the appropriate positive and negative cancer cell types. After conjugating various primary antibodies to Q-Dots, and reducing non-specific binding, we determined that DyLight conjugated antibodies allow better distinction between positive and negative expression of the marker than Q-Dots. DyLights 488 and 594 were successfully applied; However, since DAPI or Hoechst occupies another excitation/emission cube this ruled out the use of DyLight 3 The use of Alexa Fluor 680-labeled secondary antibody was much more effective than DyLight 650. Since we had no success with DyLights in the near IR range, this ruled out the simultaneous use of a fourth marker/fluorophore pair. Our primary antibody/fluorescent antibody pairs provided a feasible test to allow for interpretable results relevant to the goal of the study and the eventual clinical application. Finally we developed a protocol, which utilizes a combination of DyLight conjugated primary antibodies and primary /secondary antibody pairs for IF evaluation of two 7.5 mL tubes of blood from each patient. PSA [7, 8] and ER [30, 31] expression have previously been examined in CTCs but not as part of this 4-marker panel for the identification of the primary site in CUP patients.

ER-negative breast carcinoma poses a particular challenge for accurate diagnosis. Thus our algorithm focused the diagnosis in ER-negative breast cancer or previously ER-positive breast cancer with loss of ER in their CTCs. The CK7-positive, TTF-1-negative, CK20-negative, Hoechst-positive signature observed in our ER-negative patient (Figure 4) is consistent with breast cancer. However, as shown in our algorithm, positive ER expression will rule out most bile duct, pancreas, esophagus, stomach, kidney and lung cancers (Figure 1). Serving as an adjunct to standard biopsy, our CTC blood test is able to support a breast cancer diagnosis even in ER-negative cases. A CTC blood test has the advantage of being minimally invasive thus enabling us to obtain a second blood sample from an ER-positive breast cancer patient who appeared to have lost all ER expression in her CK7-positive/Hoechst-positive CTCs. However, results from the second on-study blood draw showed 21.7% of the CK7-positive/Hoechst-positive cells demonstrated ER expression. Since only a small percentage of breast cancer cells need to be ER-positive (or PR-positive) for the breast cancer to be hormone-receptor positive, it is not surprising that an earlier sampling of circulating tumor cells yielded ER- negative CTCs. Nevertheless, the CTCs from the two blood draws likely demonstrate dynamic intratumoral heterogeneity. Theoretically CTCs are released from either the primary tumor or the metastases. As this patient's primary tumor was removed prior to both on-study blood draws, it would be interesting to compare CTC ER expression with that from the metastatic site. Nevertheless the presence of ER in the CTCs from the second on-study blood draw enabled us to make a more accurate assessment. A lack of ER expression in CTCs from patients with ER-positive primary breast cancer has been previously reported [30, 31] The presence of ER-negative CTCs in ER-positive cancer patients is significant as these CTCs may escape ER-targeted therapies, conferring selection pressure for their survival. This is a possibility for our second breast cancer patient with ER-positive primary tumor with aromatase inhibitors as part of her therapy regimen.

The presence of ER-positive, Hoechst-positive, CK7-negative cells in the blood of the second breast cancer patient could be interpreted as CK7 loss in CTCs. Only a subset of cytokeratins are down regulated during epithelial to mesenchymal transition (EMT). There is a dearth of information on the frequency of CK7 loss in breast cancer CTCs, where antibody cocktails targeting several cytokeratins are often utilized [32, 33]. Although we may speculate loss of more broadly expressed epithelial markers to occur more frequently during EMT, CK7, which is positive in upper gastrointestinal, respiratory and gynecological malignancies [18, 21] could potentially be lost during EMT in breast cancer CTCs.

Our prostate cancer patient demonstrated a CK7-positive, TTF-1-negative, PSA-positive profile in Hoechst-positive CTCs which can only be diagnosed as prostate cancer. Moreover, PSA marker expression has been shown to be stable after metastasis in prostate cancer patients and no metastatic tumors of non-prostatic origin express PSA in this study [34]. CK7-positivity in prostate cancer has been reported [28].

A third tube of blood from each patient is used for FDA-cleared CellSearch analysis of the CTCs using the epithelial kit to enumerate the CTCs and diagnose carcinoma. Although our initial approach was to further immunophenotype the same cells captured by the CellSearch using 4 of the 5 markers, it was determined that both the FMSA and CellSieve enable superior IF analysis of cells captured on the device. It is important to note that we obtained comparable or greater numbers of CTCs using these size-based approaches as with FDA-cleared CellSearch. A broader limitation of the utilization of EpCAM for CTC selection is that many of the CTCs may not express EpCAM. Cells undergoing EMT and poorly differentiated stem cell-like tumor cells are likely EpCAM negative. Our study identified metastasis of the four most common carcinomas (prostate, lung, breast and colon) from CTCs, which typically express high levels of EpCAM [33]. Nevertheless, if any of the CTCs have lost EpCAM, CK8, CK18, or CK19, our size-based CTC enrichment-capture approaches will enable us to capture these cells, which would otherwise escape detection by CellSearch.

CUP can have early [10] and unusually aggressive metastatic dissemination without readily identifiable primary tumor [9]. It is presumed that before the primary tumor becomes large enough to be readily detectable, the tumor has already metastasized. The primary tumor may either be slow growing or have possibly involuted [21]. Developing novel techniques to determine the site of origin in CUP patients is crucial, since these patients who remain to have unknown primary after conventional detection method carry a poor prognosis and often have delay in treatment due to lack of a definitive diagnosis. Moreover, prognosis and treatment of metastatic adenocarcinomas is tied to the primary tumor. CUP patients whose primary cancer cannot be identified are usually treated with empirical platinum-based chemotherapy [9], which is often not as effective as treatments tailored to specific sites. This assay is being developed due to the less specific and often delayed diagnosis in current practice when evaluating CUP. Studies have shown that in 20 to 50% of patients, a primary site is never identified [9]. Using CTCs in this setting may improve the detection of this poorly understood cancer and it also has the benefit of being largely non-invasive. However, some tumors are so poorly differentiated that they may still be left without a clear diagnosis whether a traditional immunohistochemistry (IHC) of the biopsy or biopsy IHC with accompanying CTC IF blood test are employed. Genomic analysis with algorithm-based markers in CTCs may provide an alternative to this protein-antibody based approach to identifying the primary site.

Our future studies involve implementation of an approved clinical study, which is divided into two phases/stages. In the first stage/phase of the CUP-CTC study, we will recover CTCs in 20 patients with metastatic cancers of known primary (lung, prostate, breast, colon), and then use our novel assay to obtain informative marker expression information to aid in the diagnostic process. The primary site of origin as determined from the novel CTC-CUP assay will be verified using classical immunofluorescence and common IHC markers as is routinely performed in the course of clinical care of patients. In second stage/phase of the CTC-CUP study, we plan to enroll 20 patients being evaluated for suspected CUP.

The results of this study will not replace the standard diagnostic evaluation including history and physical exam, laboratory testing, imaging, tissue biopsy and clinical correlation; however, we aim to use the knowledge obtained to improve upon the accuracy and timing of diagnosing the tissue of origin in patients with CUP in a noninvasive manner in the future. Patients with poorly differentiated carcinomas may pose the greatest challenge in identifying the primary both in terms of biopsy of the metastatic site(s) and the CTCs. If this small clinical study is successful, further implementation will require prospective clinical trials. We estimate that if a simple CUP-CTC blood test can facilitate diagnosis in as few as 10-20% of patients with CUP, such relatively noninvasive and inexpensive assay would have wide appeal for clinical use especially in the outpatient setting.

## MATERIALS AND METHODS

### Antibodies

The following antibodies and fluorophores were used: CK7 (Dako), CK20 (Dako), TTF1 (Invitrogen), ER clone 16660 (Abcam), PSA (Cell Signaling) and AFP (Cell Marque). DyLight 594, DyLight 488 (Pierce), Q-Dot 605 and Q-Dot 655 (Life Technologies).

### CTC capture and Immunophenotyping

Blood (∼7.5mL) collected in an EDTA tube was filtered through a single FMSA device within 4hrs of collection. The device was gently moved to a glass microscope slide and fixed with Cytofix/Cytoperm (BD Biosciences) for 30-45 minutes at 4 degrees Celsius, washed in PBS (phosphate buffered saline) and incubated in PBS with 0.3% Triton-X-100/0.1% BSA (modified PBT) for 10 minutes. The samples were subsequently blocked for > 1hr with 1:1 mixture of serum from the same species as the secondary antibody and modified PBT. The first set of antibodies are diluted in modified PBT overnight at 4 degrees Celsius in a humidified chamber with paraffin covering the device resting on a glass slide. After washing with PBS, the secondary antibody diluted in PBT was added at RT for 2hrs. After gentle washing with PBT and PBS, the device was either washed with deionized water for nuclear staining or incubated with conjugated antibody/antibodies diluted in PBT for 5.5 to 6hr incubation at 4 degrees Celsius followed by washing in PBT, PBS and deionized water. DAPI or Hoechst freshly diluted in deionized water was added for 1 minute and washed with deionized water. After adding mounting media and a coverslip, the slides were viewed. The same protocol was used for the CellSieve (Creatv Microtech) device, which may be retained longer in the filter holder assembly before moving it to a glass slide. The CellSieve device (7micron pore diameter) is used with a syringe pump for gently pushing the blood through the device and washing with PBS.

Plated cells positive and negative for all the markers were also evaluated for the presence of the marker proteins on the same day as the patient sample for the metastatic ER/PR positive breast cancer patient on both days that blood was drawn for CTC enrichment. MCF7 (ER positive control) and SKBR3 (CK7 positive control) cells were spiked into normal donor blood and captured on the CellSieve device the same day as the patient sample for this patient's first on-study blood draw. The antibodies were diluted for the positive/negative control plated cells, spiked positive controls and patient samples in one tube before aliquoting to ensure that results from patient samples cannot be attributed to the experimental execution.

Blood (7.5mL) collected in a CellSave tube was used run through the Janssen (Veridex) CellSearch system using an EpCAM-based Circulating Epithelial Cell Kit within 72hrs of collection.

Post-CellSearch immunofluorescence was performed after collecting cells by flushing the CellSearch cassette with PBS and cytospin of the cells onto a glass slide.

### Imaging

Imaging was primarily performed on a Nikon Eclipse T*i* inverted microscope and NIS Elements software or to evaluate Q-dots CRi Nuance software for multispectral imaging (El-Deiry laboratory). Some automated imaging was conducted using a DeltaVision Elite microscope system, plus an upright fluorescence microscope with a Retiga Exi high-speed CCD camera with QED software for image acquisition and Huygens software (Penn State Hershey Imaging Core).

## SUPPLEMENTARY MATERIAL FIGURES


