# Supplementary material for: A multiplexed marker-based algorithm for diagnosis of carcinoma of unknown primary using circulating tumor cells
Source: Oncotarget. 2015 Dec 18;7(4):3662–76. doi: 10.18632/oncotarget.6657 (PMC4826160; doi:10.18632/oncotarget.6657)
Supplement: Supplementary file 1 [file oncotarget-07-3662-s001.pdf]

A multiplexed marker-based algorithm for diagnosis of carcinoma of unknown primary using circulating tumor cells

Supplementary Material

FIGURE S1. Expanded set of markers for carcinoma identification.

FIGURE S2. Cytokeratin 7 (CK7) expression in SKBR3 cells spiked into blood.

FIGURE S3. Cytokeratin 20 (CK20) marker validation.

FIGURE S4. Estrogen receptor (ER) marker validation.

FIGURE S5. Prostate specific antigen (PSA) marker validation.

FIGURE S6. Thyroid transcription factor 1 (TTF1) marker validation.

FIGURE S7. Alpha fetoprotein (AFP) marker validation.

FIGURE S8. Carcinoembryonic antigen (CEA) marker validation.

FIGURE S9. Carcinoembryonic antigen: reduction of non-specific binding.

FIGURE S10. ER-negative metastatic breast cancer patient: Experimental schematic.

FIGURE S11. Metastatic breast cancer patient with elevated CTC count: Second immunofluorescence (IF).

FIGURE S12. Metastatic breast cancer patient: cytokeratin 7, estrogen receptor and phase contrast microscopy.

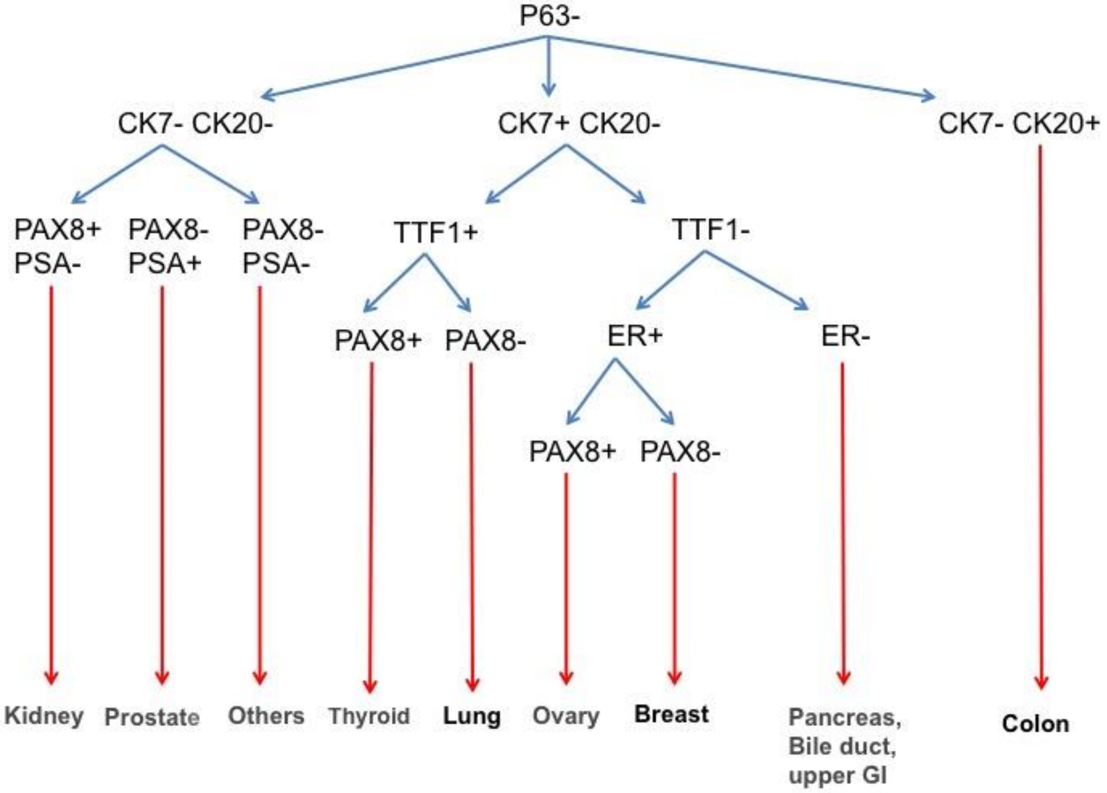

FIGURE S1. Expanded set of markers for carcinoma identification. A full spectrum of carcinomas are identified by immunofluorescence determination of the presence or absence of each of five out of six marker proteins in p63 negative cancers. Cytokeratin 7 (CK7), cytokeratin 20 (CK20), thyroid transcription factor 1 (TTF-1), and Pax-8 with estrogen receptor (ER) in females and prostate specific antigen (PSA) in males.

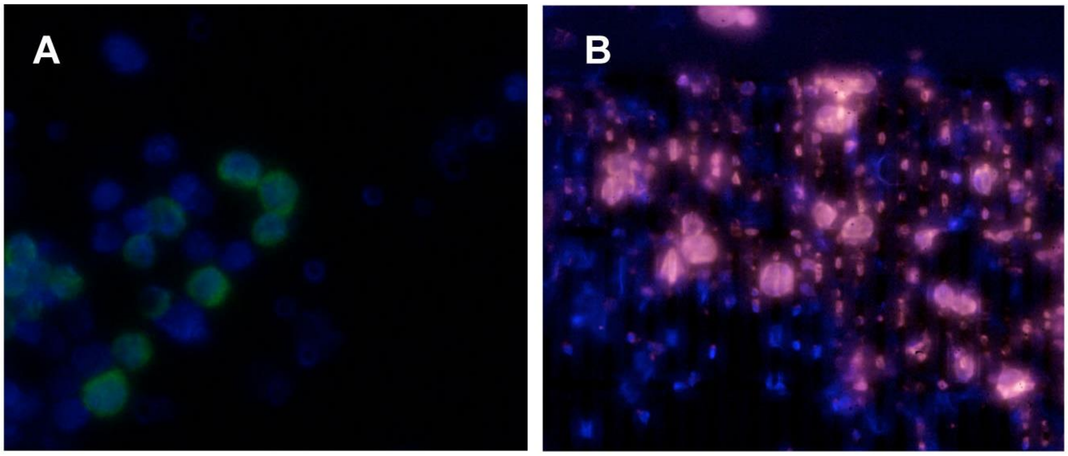

FIGURE S2. Cytokeratin 7 (CK7) expression in SKBR3 cells spiked into blood. A) Creatv Micro Tech CellSieve device. CK7 (green) and nuclear staining (blue). Zoomed B) FMSA device. CK7 conjugated to Q-dot 605 (pink) and nuclear staining (blue).

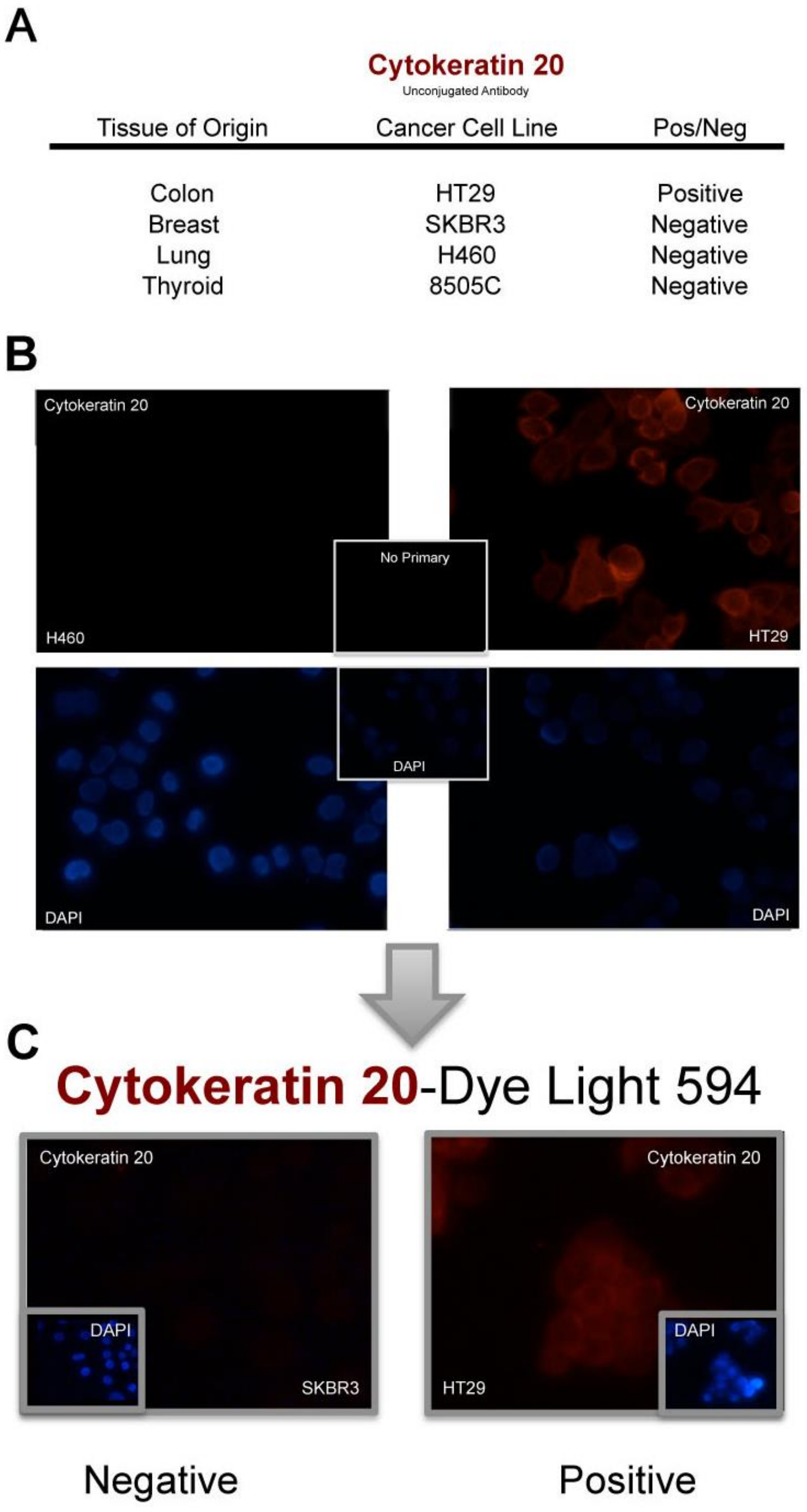

FIGURE S3 Cytokeratin 20 (CK20) marker validation. CK20 demonstrates positive expression in HT29 colon cancer cells but negative expression in H460 lung cancer

cells and SKBR3 breast cancer cells. A) CK20 expression in all the positive and negative cell lines tested with unconjugated primary antibody and fluorescent secondary antibody. A) Unconjugated CK20 antibody. 4',6-diamidino-2-phenylindole (DAPI) nuclear staining (bottom panels) B) DyLight 594 conjugated CK20 antibody. DAPI nuclear staining (insert). Zoomed image.

A

Estrogen Receptor

Unconjugated Antibody

Tissue of Origin	Cancer Cell Line	Pos/Neg
Breast	MCF7	Positive
Breast	SKBR3	Negative
Fibroblasts	MRC5	Negative
Lung	H460	Negative
White blood cells	White blood cells	Negative

B

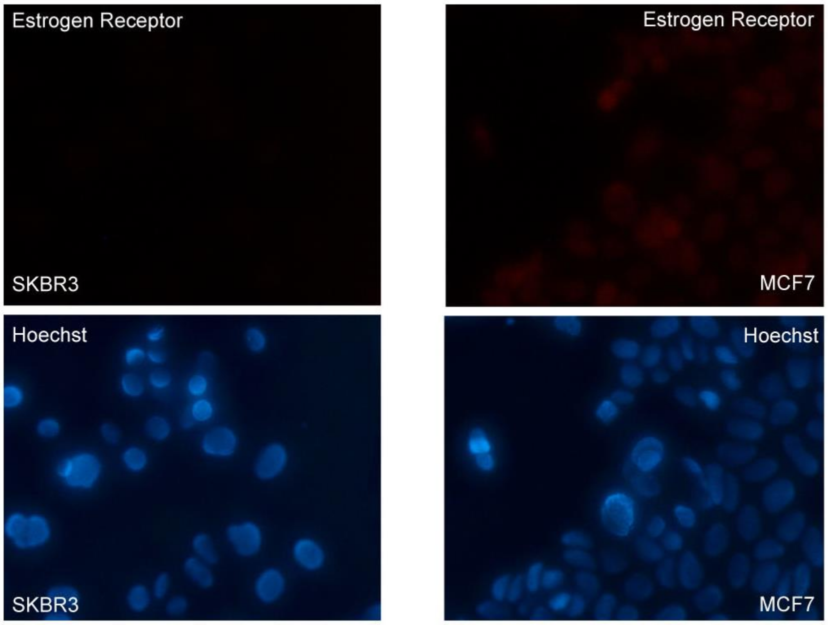

FIGURE S4. **Estrogen receptor (ER) marker validation.** ER demonstrates positive expression in ER-positive MCF7 breast cancer cell line but negative expression in ER-negative SKBR3 breast cancer cells. A) ER expression in all the positive and negative cell lines tested with unconjugated primary antibody and fluorescent secondary antibody. A) ER-positive and ER-negative breast cancer cells evaluated with unconjugated ER antibody and fluorescent secondary antibody (top panels). 4',6-diamidino-2-phenylindole (DAPI) nuclear staining (bottom panels).

A

Prostate Specific Antigen

Unconjugated Antibody

Tissue of Origin	Cancer Cell Line	Pos/Neg
Prostate	LnCAP	Positive
Prostate	DU145	Negative

B

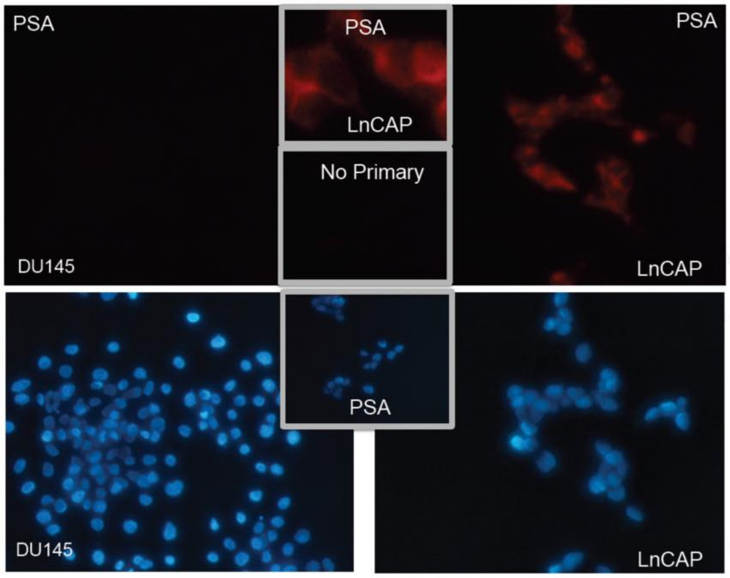

C

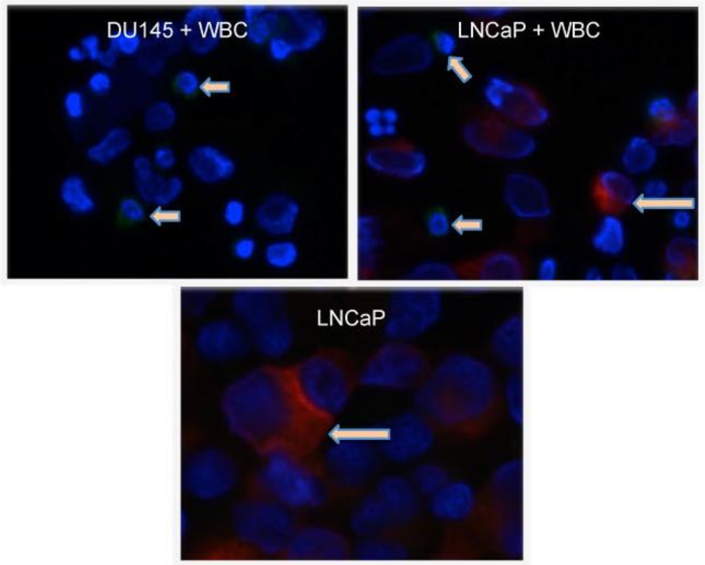

FIGURE S5. **Prostate specific antigen (PSA) marker validation**. A) PSA expression in cell lines tested. B) PSA expression is seen in PSA-positive LnCAP prostate cancer cells but not in PSA-negative DU145 prostate cancer cells. 4',6-diamidino-2-phenylindole (DAPI) nuclear staining of cells in the same fields are shown in the bottom panels. C) PSA expression (red; PSA primary and Cy3 secondary antibody; long arrows) in DU145 cells or LnCAP spiked into white blood cells (wbc) from normal donor blood (top panels) and LnCAP cells alone (bottom panel). Leukocyte common antigen (CD45 from Cell Signaling; green, Alexa Fluor 488 secondary antibody) staining of WBCs (short arrows). Zoom area of 600x image.

A

Thyroid Transcription Factor-1

Unconjugated Antibody

Tissue of Origin	Cancer Cell Line	Pos/Neg
Lung	H441	Positive
Lung	H460	Negative
Normal fibroblasts	HFF, MRC-5	Negative
Colon	HT29	Negative
Breast	SKBR3	Negative
White blood cells	White blood cells	Negative

B

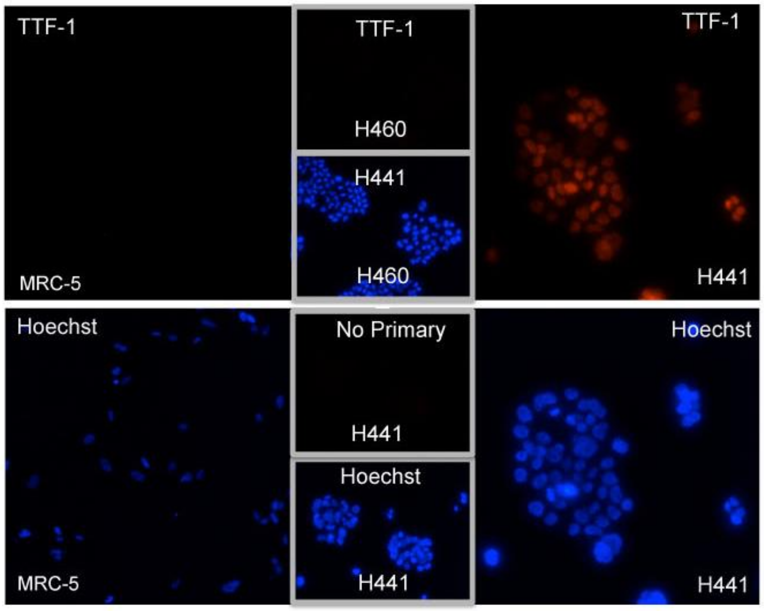

C

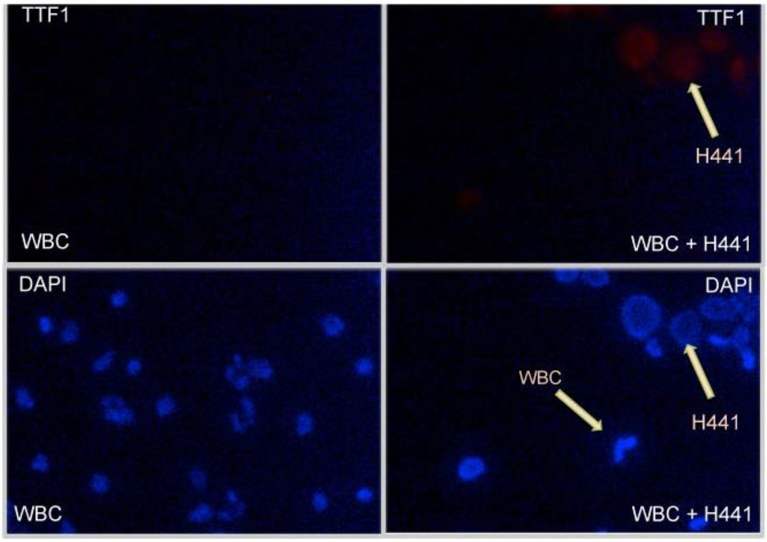

FIGURE S6. **Thyroid transcription factor 1 (TTF1) marker validation**. A) TTF1 expression in cell lines tested. B) TTF1 is expressed in H441 lung cancer cells but not MRC5 fibroblasts. 4',6-diamidino-2-phenylindole (DAPI) nuclear staining of cells in the same fields are shown in the bottom panels. C) TTF1 is not expressed in cytospun white blood cells from normal donor blood. TTF1 is selectively expressed in H441 lung cancer cells spiked into white blood cells from normal donor blood and cytospun. DAPI is shown in lower panels.

A

Alpha fetoprotein

Unconjugated Antibody

Tissue of Origin	Cancer Cell Line	Pos/Neg
Liver	HepG2	Positive
Breast	SKBR3	Negative
Thyroid	8505C	Negative

B

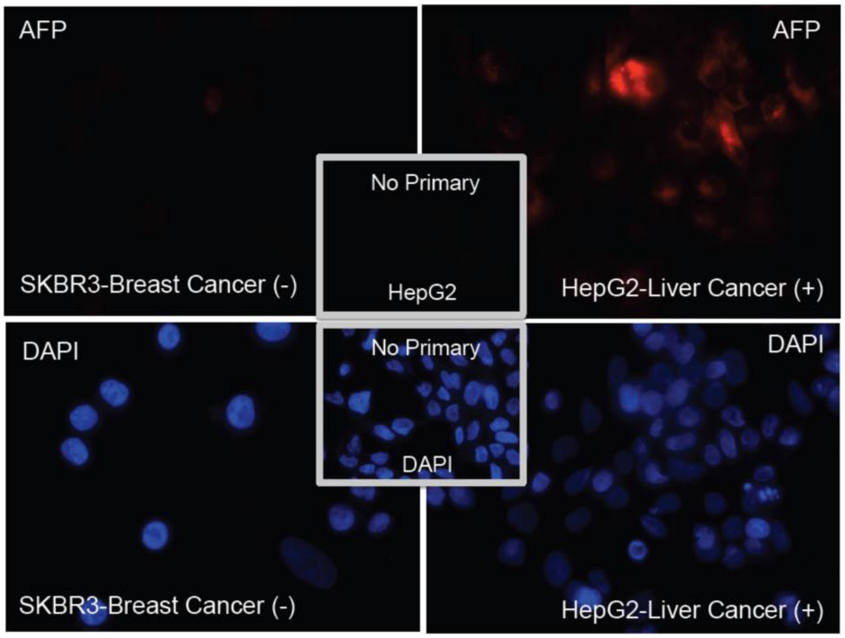

FIGURE S7. Alpha-fetoprotein (AFP) marker validation. A) AFP as an unconjugated primary antibody was evaluated in positive and negative cancer cell types. B) AFP expression after incubation with unconjugated primary antibody, followed by fluorescent secondary antibody (red, left panels). 4',6-diamidino-2-phenylindole (DAPI) staining identifies the nucleus in the same field (blue, bottom panels).

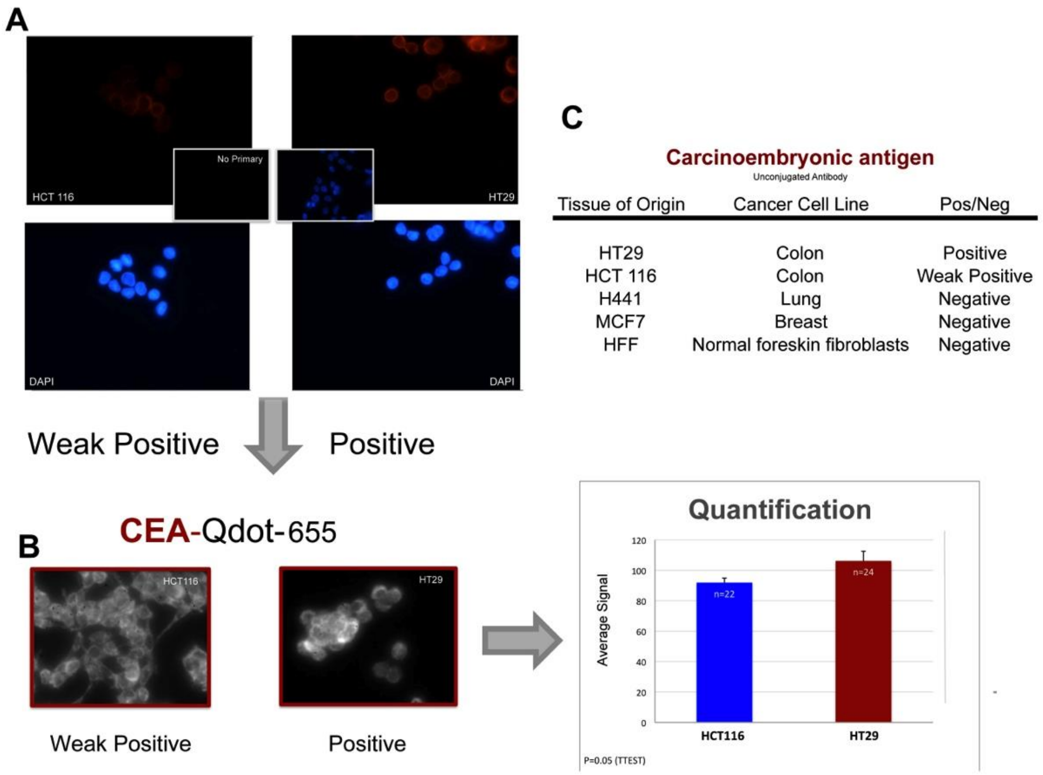

FIGURE S8. Carcinoembryonic antigen (CEA) marker validation. CEA demonstrates positive expression in HT29 cells but weak positive expression in HCT 116 cells. A) Unconjugated CEA antibody. B) Q-Dot 605 conjugated CEA antibody. Quantification: Regions of interest (22 to 24) around cells was utilized to calculate average signal intensity. C) Colon cancer lines tested for weak positive and positive CEA expression.

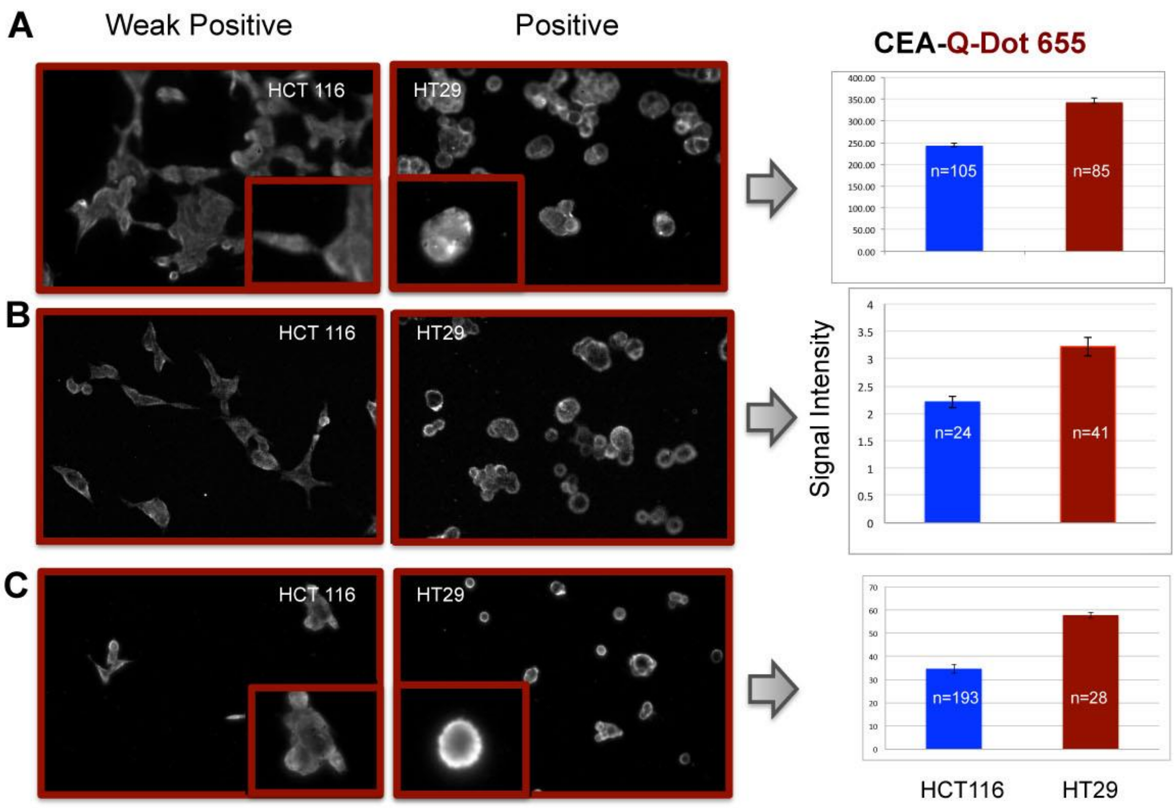

FIGURE S9. Carcinoembryonic antigen: reduction of non-specific binding. Three different approaches to reduce non-specific binding of Q-dot 655 conjugated CEA all incorporated reduction of antibody incubation time. A) Block with 3.75% milk in TTBS, B) 6% BSA for blocking and in antibody diluent C) 1:700 free inactivated Q-Dot 800 incubated with CEA antibody conjugated to Q-Dot 655.

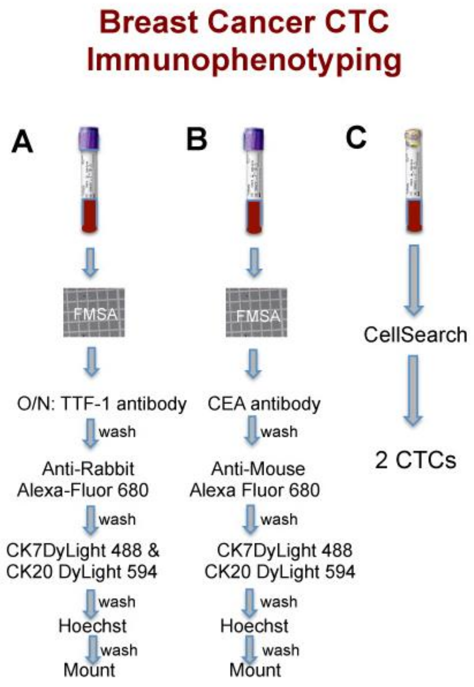

FIGURE S10. ER-negative metastatic breast cancer patient: Experimental schematic. The ER-negative metastatic breast cancer patient ‘s CTCs were captured and subject to the immunofluorescence protocol as shown. The results from tube A are shown in Figure 5.

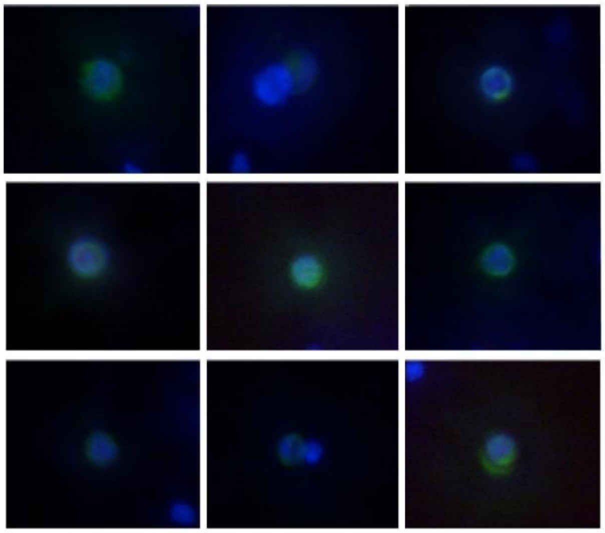

FIGURE S11. Metastatic breast cancer patient with elevated CTC count: Second immunofluorescence (IF). A metastatic Estrogen receptor (ER)/Progesterone receptor positive patient IF blood draw 26 days after the first IF blood draw. High power images of 9 additional CTCs captured with the CellSieve device not previously shown in Figure 9 or Figure 10. Merged images of Cytokeratin 7/Alexa Fluor 488 (green), ER/Cy3 (red) and Hoechst 33342 nuclear stain (blue). Experimental protocol shown in Figure 9B: Results from tube A are shown above and this patient had 46 CTCs by CellSearch (tube C, Figure 9) during this blood draw.

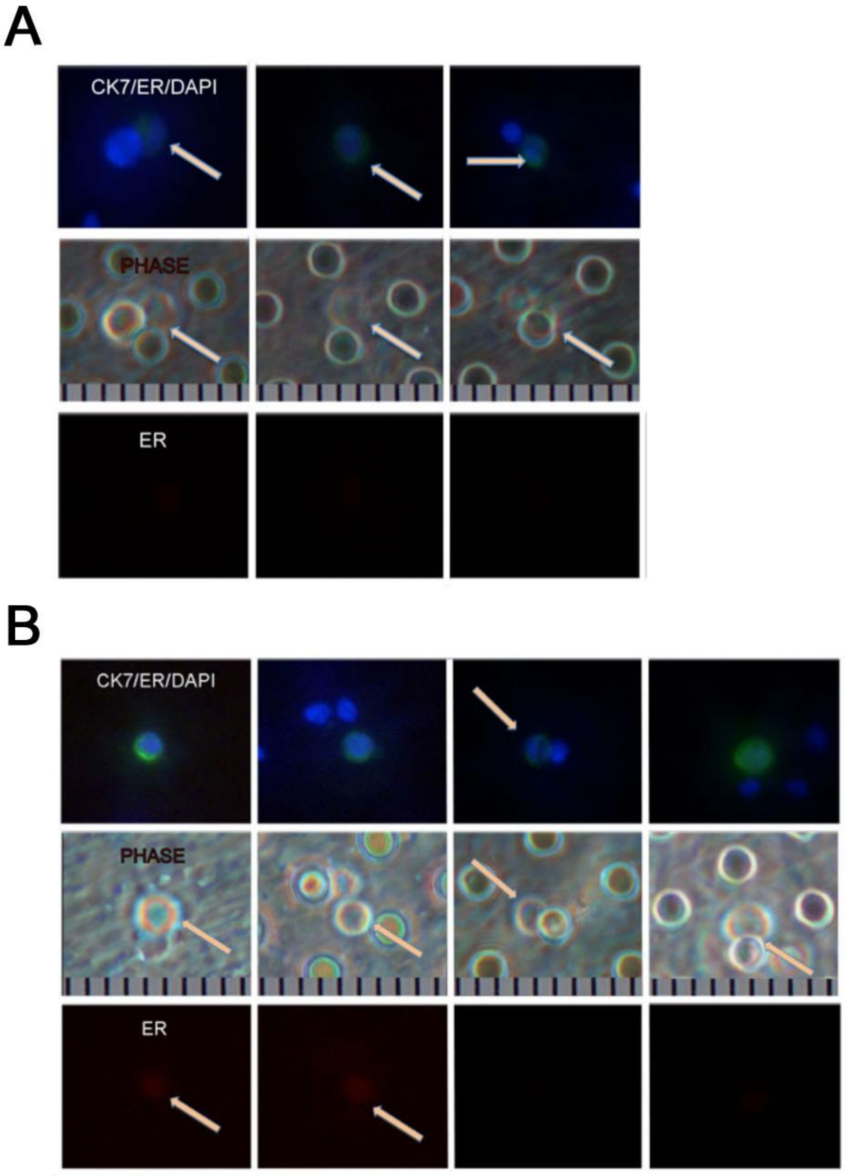

FIGURE S12. Metastatic breast cancer patient: cytokeratin 7, estrogen receptor and phase microscopy. A metastatic Estrogen receptor (ER)/Progesterone receptor positive patient IF blood draw 26 days after the first IF blood draw. High power images of 3 different CTCs (Panel A) and 4 different CTCs (Panel B) not previously shown in Figures 9, 10 and Supplementary Figure 9. Top row of each panel: Merged images of

Cytokeratin 7 (green), Cy3 (red) and Hoechst 33342 nuclear stain (blue). Middle row of each panel: Phase microscopy of the same field as the panel above. Arrows point to phase images of cells that show CK7/Hoechst expression with variable ER expression in the image above. Bottom row: ER expression alone of the same field as the images above. Experimental protocol is shown in Figure 9B: Results from tube A are shown above and this patient had 46 CTCs by CellSearch (tube C; Figure 9B) during this blood draw.
